# Redefining intelligence: collaborative tinkering of healthcare professionals and algorithms as hybrid entity in public healthcare decision-making

**DOI:** 10.1007/s00146-024-02177-7

**Published:** 2025-01-09

**Authors:** Roanne van Voorst

**Affiliations:** Department of Anthropology, University of Amsterdam, Amsterdam, Netherlands

**Keywords:** Human–nonhuman decision-making, Clinical decision-making, AI, Ethical AI, Tinkering

## Abstract

This paper analyzes the collaboration between healthcare professionals and algorithms in making decisions within the realm of public healthcare. By extending the concept of ‘tinkering’ from previous research conducted by philosopher Mol (Care in practice. On tinkering in clinics, homes and farms Verlag, Amsterdam, 2010) and anthropologist Pols (Health Care Anal 18: 374–388, 2009), who highlighted the improvisational and adaptive practices of healthcare professionals, this paper reveals that in the context of digitalizing healthcare, both professionals and algorithms engage in what I call ‘collaborative tinkering’ as they navigate the intricate and unpredictable nature of healthcare situations together. The paper draws upon an idea that is increasingly common in academic literature, namely that healthcare professionals and the algorithms they use can form a hybrid decision-making entity, challenging the conventional notion of agency and intelligence as being exclusively confined to individual humans or machines. Drawing upon an international, ethnographic study conducted in different hospitals around the world, the paper describes empirically how humans and algorithms come to decisions together, making explicit how, in the practice of daily work, agency and intelligence are distributed among a range of actors, including humans, technologies, knowledge resources, and the spaces where they interact. The concept of collaborative tinkering helps to make explicit how both healthcare professionals and algorithms engage in adaptive improvisation. This exploration not only enriches the understanding of collaborative dynamics between humans and AI but also problematizes the individualistic conception of AI that still exists in regulatory frameworks. By introducing empirical specificity through ethnographic insights and employing an anthropological perspective, the paper calls for a critical reassessment of current ethical and policy frameworks governing human–AI collaboration in healthcare, thereby illuminating direct implications for the future of AI ethics in medical practice.

## Introduction

The cardiologist’s gaze was firmly fixed on the computer screen, displaying a fluctuating line with red segments. The red segments, the doctor explained, represented patients about whom he should have grave concerns. They were heart patients who, based on their self-reported data, had not taken their prescribed medication, or patients who, for other reasons, were at relatively high and acute risk. Artificial Intelligence (AI) had calculated their data, which was then presented in a visually accessible manner to clinicians working in this ward. The algorithm succinctly illustrated what needed to be done: those patients marked in red as high-risk needed to be called this very morning by the cardiologist. “But obviously, that doesn't necessarily mean I will,” she said, shrugging her shoulders. “The algorithm suggests one thing to us doctors, and I arrive at my own idea in my own way, and I guess you can say we negotiate, me and my digital colleague here, until we reach a sound decision together.”

This paper examines the collaborative decision-making processes involving both algorithmic technologies and the individuals who work with them, thus influencing them through their actions, data inputs, evaluations and feedback. As Laura Savolainen and Minna Ruckenstein have recently argued, this feedback loop between man and machine underscores the challenges of identifying agency within hybrid, distributed, and mutually reinforcing systems: “Both human and machine can begin to self-correct and modify in response to the other’s signals and stimuli, as if acting in unison”(2024: 3074).

This paper focuses on the specific workings of such unisons within the context of the digitizing public healthcare sector. Drawing upon an international ethnographic study conducted in four hospitals, the paper illustrates how the well-known premise that agency and intelligence are distributed among various actors—including humans, technologies, knowledge resources, and the environments in which they operate—manifests in the daily work of professional caregivers collaborating with AI systems. The paper discusses two concrete case studies, which both complicate the notion of human in the loop, each in a very different way. In the first case, there appears to be a genuine unison between physicians and AI, as they together arrive at diagnoses by means of collaboration, intuition and what Pols has called a ‘mechanical sixth sense’. Here, humans are clearly in the loop, but it would be false to assume they make the decisions alone, or separated from the technology. In the second case, although humans are formally acting as overseers of AI and are aware of their responsibilities, their daily collaboration is undermined by naïve trust in the system and a lack of understanding of how it operates. Consequently, their own critical involvement in the outcome is minimal. Both cases challenge the assumptions present in legislation surrounding AI and healthcare, as I will demonstrate below. Within this context, there often persists an idea—which has already been criticized in academic literature—that intelligence is exclusively confined to individual humans, or machines. It is assumed that a human must—and can—intervene as an overseer, whenever an AI makes an error. This paper shows that this assumption is not only frequently incorrect but can also lead to dangerous ethical consequences.

The paper builds on two streams of literature: the first concerns the aforementioned, established idea that the intelligence in AI should not be viewed as solely belonging to humans or machines, but rather as a fluid partnership between human and technical components in complex social and computational environments. This idea is further discussed in the literature review. Furthermore, the paper also contributes to another stream of literature, particularly prominent in the field of Medical Anthropology. This literature describes how doctors and nurses utilize technology, and how they do so in a manner that, according to them, best leads to what they consider as ‘good care’. Much of this work demonstrates that medical professionals do not simply follow medical protocols but also rely on their intuition and improvisation to ultimately make decisions they deem appropriate for their patients. Similarly, anthropologists have shown that professional caretakers often work with newly introduced technology in ways that do not align with the user manual or the intended use by the technology’s designers. The work of professional caretakers is characterized by constant adaptation to the context and moment. In Medical Anthropology, this has become known as ‘tinkering’, and one of such tinkering practices has been described for professional caretakers developing a ‘mechanical sixth sense’ in their work with technology. I find these concepts very helpful for understanding how professional caretakers strive to deliver “good care” to their patients in an era of technologization and digitalization, yet they have not yet been applied to AI. This is unfortunate because the collaboration between healthcare providers and AI presents an interesting example of how improvisation, adaptation, and intuitive adjustments are constantly made in the quest for diagnoses and decisions. Therefore, this paper introduces the concept of ‘collaborative tinkering’. By building upon the concept of “tinkering” from previous studies conducted by philosopher Mol (2010) and anthropologist Pols (2009)—who emphasized the improvisational and adaptive practices of healthcare professionals—this paper reveals that both professionals and algorithms, together as one hybrid unit, engage in collective tinkering as they collaboratively navigate the complex and unpredictable nature of healthcare situations. Acting in unison, they both constantly respond to each other’s feedback and stimuli. Hence, this article broadens the concept of tinkering to encompass situations where algorithms, in continuous interaction with the ‘human in the loop’ (the healthcare professional who assesses and interprets the algorithm’s outputs), tinker collectively, as a unified hybrid entity. The main objective of this paper is to prompt a re-evaluation of how we comprehend and ethically respond to AI in the contemporary world. By further challenging the notion of the individuality (Lenartowicz et al. 2016; Simondon 1992) of AI, which, as I will argue, is still prevalent in AI regulation, the paper raises new questions concerning the ethics of human–nonhuman decision-making and advocates for a re-examination of AI ethics.

## Literature review

### Human–nonhuman decision-making in healthcare, and the problem with ‘ethical AI’

By governments worldwide, big data and AI systems are presented as inevitable for the future of healthcare. Research has shown that datasets in AI (re)produce social biases, discriminate, and limit personal autonomy. Automated decision-making systems overwhelmingly tend to punish the poor and middle-class through mechanisms of control (Eubanks 2018; Passchier 2021). As a response to this alarming research, public and scholarly concerns about algorithmic ethics have risen. Governments and tech companies have extensively endorsed legal frameworks and regulatory guidelines for socalled ‘ethical’, ‘responsible’, or ‘fair’ AI—hundreds of these frameworks now exist worldwide. The specific regulations vary from one framework to another, but a crucial aspect common to all—and particularly significant for this research project—is ensuring that algorithms remain explainable so that software programmers, and ideally also the software users, can understand how decisions are reached. The oversight role of humans is another necessary condition for labelling AI as “ethical.” The idea is that the algorithm provides calculations or advice to the human physician, who then decides whether to follow or deviate from that advice (Zanzotto 2019). Elsewhere, I have argued that the notion of human oversight is naïve in the context of public healthcare, primarily discussing issues related to the lack of effective AI training for caretakers—who are specialized in medicine, not programming—as well as the unrealistic expectations placed upon them to keep up with AI developments, while many are already overburdened with work (van Voorst 2024). Other scholars have suggested that these problems also persist in what is currently labeled as “explainable” or transparent AI. For example, in a concerning article in The Lancet, Ghassemi, Oakden-Raymer, and Beam (2021) argue that current explainability methods cannot provide clear and reliable explanations for each individual decision made by the AI system. Other scholars, too, express concerns over the effectiveness of ‘ethical AI’ regulations for yet different reasons (c.f. Tigard 2019). They caution that laws and guidelines alone are insufficient to ensure ethical AI practices; what truly matters is how people use AI in practice. This is why applying an anthropological perspective, with its focus on on-the-ground practices studied through ethnographic fieldwork, is a highly suitable approach to examine this highly debated issue; this paper is based on such anthropological research, conducted in hospital settings where professional caretakers make decisions in collaboration with algorithms.

There has not been extensive empirical research demonstrating what this human–nonhuman collaboration looks like in hospitals and healthcare settings on a daily basis. However, research in other work environments where humans and machines make decisions together suggests that the assumption that incorporating oversight and override will automatically result in actual human intervention is based on limited empirical evidence. Studies in other, more extensively examined fields, such as predictive policing and criminal justice, indicate that human supervisors of AI systems often neglect to scrutinize algorithms and rarely exercise their own judgement (Hannah-Moffat 2013; Monahan and Skeem 2016; Peeters 2020;), partly because algorithms are so complex to comprehend, hindering human oversight (Harcourt 2007; Janssen and Van den Hoven 2015; Pasquale 2015), or due to the continual algorithmic adjustments in machine learning impeding human oversight (Danaher 2016; Binns 2018). This knowledge is pertinent to this paper for two reasons. First, it demonstrates that we still lack a sufficient understanding of how the collaboration between humans and machines unfolds in everyday life and work. Even when a human overseer is assumed to be able to override the algorithm's advice when necessary, this does not necessarily mean that the human will actually intervene. Not everything that is theoretically possible or legally permissible occurs in practice, for reasons already mentioned above (Brown and van Voorst 2024). This suggests not only that there is much room for debate on what is currently considered “ethical” or “humane” AI, but it also highlights the need for practical examples that can illustrate whether and why regulation may or may not be effective. Second, and most relevant to this paper, it becomes apparent that in these ethical regulatory frameworks, a strict categorization between humans and machines still exists, despite the fact that this idea has long been criticized by scholars. Already in 1992, Simondon wrote that individuals should not be regarded as static, well-defined Aristotelian entities with pre-established characteristics. Instead, they should be viewed as fluid beings in a state of constant evolution and transformation. This perspective has important consequences for how we consider human decision-making. Humans are not autonomous in making decisions; rather, their choices and identities are shaped by ongoing interactions with their environments and the technologies they engage with, reflecting a more complex and dynamic understanding of human agency in contemporary society. Several years later, Michel Callon and John Law would write that agency is always distributed between humans and the nonhuman actors with whom they interact. They discussed the ‘hybrid collectif’ (1995: 481)—an idea that became commonplace in Science and Technology Studies (STS) and anthropology. For example, Hayles (2022) uses the example of human–machine hybrid reading, such as reading an e-book, to argue that this activity should be viewed as a cognitive assemblage in which information, interpretations, and meanings circulate between humans who read and machines that read. She suggests that such a spatial arrangement can be best described as “cognitive assemblages”: collectives consisting of humans, nonhumans, and computational media in which cognition, agency, and intentionality are distributed among multiple actors and agents (Hayles 2022: 1195). In her work “Unthought” (2017), Hayles expands on her concept of cognition, urging readers to acknowledge the cognitive capabilities of technical systems, including their ability to make choices, decisions, and predictions of future scenarios—similar to the functions of Large Language Models. As Louise Amoore (2019: 4) notes, these algorithmic practices are increasingly influencing the politics and ethics of our contemporary world. Going beyond these pressing issues, Hayles challenges the traditional human/nonhuman binary that persists in academic and public discourse, proposing an alternative distinction: cognizers versus noncognizers. On one side are humans, all other biological life forms, and many technical systems, while on the other side are material processes and inanimate objects (Hayles 2017: 30).The evolution of humans and algorithms together is creating novel ways of reading and cognition that defy traditional classifications of man and machine. Hayles’ perspective resonates with feminist scholarship on science and technology, such as the work of Donna Haraway (cf. 1991), and more notably, Actor-Network Theory (ANT) developed by aforementioned Michel Callon and Bruno Latour. ANT emphasizes the interconnectedness of human and non-human actors, including technologies, and the concept of distributed agency aligns well with its core principles. By focusing on the interactions and relationships between various actors, including both human and technological entities, a comprehensive approach to ethics and politics within technological systems is advocated. The notion that intelligence is dispersed among different actors, rather than being confined to individuals, also mirrors the ANT idea of non-hierarchical networks of actors working together.

Building on these ideas, Siles (2023) describes how people ‘enact’ algorithms, exploring how users and recommendation algorithms on platforms engage in a continuous process of adaptation, reflecting the ways in which users modify their behaviors in response to algorithmic suggestions. This perspective seems inspired by Annemarie Mol’s “practical ontology” (Gad et al. 2015), which assumes that actors do not act on pre-given objects, but rather bring them into being—a process she calls “enactment.” Consequently, objects acted on in many different ways become “multiples”: “more than one and less than many” (Mol 2002: 82). A “culture,” for instance, is not one coherent thing, nor is it a set of disparate things, such that every person enacts and imagines their own in isolation. Siles and colleagues propose the concept of mutual domestication to describe such dynamics in the context of algorithms: “Users incorporate algorithmic recommendations into everyday life as much as the platform works to colonize users and turn them into ideal consumers through its algorithms” (Siles et al 2019: 500). In turn, these algorithms learn from user interactions, creating a feedback loop that can lead to the refinement of both user preferences and algorithmic outputs. Similarly, Bonini and Trere (2024) theorize a symbiotic relationship between platform structural constraints and algorithmic agency, namely ‘the reflexive ability of humans to exercise power over the “outcome” of an algorithm’ (2024: 20). They utilize the classical notion of “mutual shaping” found in to examine how algorithms interact with social movements and user practices. They argue that users not only adapt to the algorithms that mediate information and public discourse but also influence the development and implementation of these algorithms in turn. Additionally, Savolainen and Ruckenstein (2024) presented the concept of “co-evolution,” describing a collaborative process wherein actions and intentions emerge from the ongoing interactions between humans and algorithms. This idea emphasizes that the relationship between humans and technology is dynamic, with each influencing and shaping the other’s behavior and goals over time. In a similar vein, De Togni et al. (2021) argue for a relational approach to understanding AI, where the location of agency itself changes to something achieved through interaction between human and machine. This also means that it makes little sense to debate whether or not AI is ‘ethical’; it is the AI in relation to the human that may be more or less ethical (2021:3). Together, these scholars convincingly argue that intelligence is always “distributed across human and technical agencies” (Amoore 2020, p. 4) and that agency is distributed over the material and the non-material (Callon and Law 1995). However, this idea has not yet resonated in ethical AI frameworks and regulations. The logic behind demands for human oversight and ‘humans-in-the-loop’ for so-called ‘ethical AI’ design is that in human–nonhuman decision-making processes, the algorithm is considered an executive apparatus that requires the intelligence of an autonomous human to act ethically. Another way to express this is that the machine, like the human, must possess a distinctly different and separate form of intelligence. The machine excels at recognizing patterns, calculating data, and making predictions based on previously inputted information, while the human can assess whether the outcomes of the algorithmic work make sense in a specific context.

To highlight the challenges posed by the regulatory focus on an imagined autonomous human individual in the context of ethical AI in hospitals, this paper analyzes empirical data from various healthcare institutions. Before presenting my research findings and methodology for engaging with human and nonhuman decision-makers in the field, it is essential to address another relevant body of work that informs this paper—one that underscores the intuitive, adaptive processes that underpin the decisions and practices of professional caretakers.

### ‘Tinkering’ and the mechanical sixth sense in healthcare contexts

When Hayles described how humans and machines collaborate to reach a conclusion she mentions that they “*feel* their way towards a solution” (1991: 6, my emphasis). She illustrates this with an example of an individual using a computer to simulate a complex nonlinear system. Instead of employing traditional mathematical methods to prove theorems, the person creates a recursive program that allows for interactive feedback. Through engaging with the computer, they develop an intuitive understanding of how the system’s display and parameters interact. The notion of intuition is significant here, as it is this very intuition that I observed during my fieldwork with professional caregivers working alongside algorithms. It also echoes the findings of philosophers and medical anthropologists Jeannette Pols and Annemarie Pols on “tinkering” and a “mechanical sixth sense”. The concept of a ‘mechanical sixth sense’, introduced by medical anthropologist Jeannette Pols in a 2010 article, is closely related to the notion of 'tinkering' that Pols and other scholars employ in their study of how professional caretakers interact with technology. While these scholars focus on technologies such as telecare rather than specifically referencing AI or algorithms, their work touches upon the intuitive and interactive approach of the hybrid human–machine decision-maker that was previously discussed in this literature review and which resonated with my own experiences during fieldwork. Mol and her colleagues (2010) have extensively explored the concept of ‘tinkering’ in the practices of professional caretakers. Tinkering can be described as an ongoing process of experimentation to determine what works in specific situations. It involves caretakers engaging in attentive and reflective experimentation to find practical solutions, constantly reinventing care practices in response to ever-changing clients and contexts. Tinkering is a dynamic and continuous process that requires close attention in research, as it is only evident in practice. This ongoing tinkering process is seen as the embodiment of what caretakers perceive as good care, encapsulating the idea of “persistent tinkering in a world full of complex ambivalence and shifting tension” (Pols 2010: 14). Pols provides a concrete example of what tinkering looks like in the context of new technology being introduced into the daily work of nurses. She analyzes Dutch care practices of nurses using telemonitoring in the care of chronic patients in the Netherlands. Many nurses expressed concerns that telecare might lead to neglect of patients and potentially hinder the development of personal relationships between nurses and patients. Pols sought to investigate ethnographically whether these concerns were justified by studying how clinical nursing practices change when telecare devices are introduced and what implications this has for notions and norms of good nursing. Interestingly, Pols found that the introduction of telecare devices did not result in the feared neglect or compromised relationships. On the contrary, she concluded that telecare led to more frequent and specialized interactions between nurses and patients. Of particular relevance to this paper is her observation that nurses developed a 'mechanical sixth sense' when working with new technology to continue providing care that they considered human, warm, and of high quality. While Pols does not provide a definitive definition or extensive description of the concept, my suggestion to develop this concept is to refer to an extra attentive state in which nurses approach the data that nowadays informs them about their patients, fearing that technology may cause them to overlook important details and potentially harm patients. It essentially describes a new way to reach the intuitive realization that a patient is not doing well. Nurses did not know before the introduction of this technology, and still do not know nowadays, exactly which senses they used, or how they could ‘know’ that a patient needed extra attention. But in the time before webcams came into homes, a sense of concern undoubtedly arose when the nurse noticed, during a home visit, that the dishes had been sitting for 6 days, or when they smelled the unwashed patient who, at two o'clock in the afternoon, was still not dressed. Webcams do not provide such detailed, multisensory impressions, so nurses had to obtain their ‘sixth sense’ in other ways: mechanical ways. By taking in a lot of data, by being extra careful to see if that data seemed reliable, by a willingness to contact the patient more often. In the daily practice of their work, this was observable by the ethnographers in Pols’ research, who noticed that the nurses are more inclined to intervene, especially when they receive data through telecare that raises concerns or doubts. They are more proactive in contacting their patients and take a proactive approach compared to before the introduction of telecare. “Ironically,” Pols notes, “the nurses may have found their mechanical sixth sense in the practice they feared would take it away: telecare practice” (2010: 383). This highlights the unexpected positive outcomes that can arise from the integration of technology into healthcare practices, challenging initial concerns and demonstrating the adaptability and resourcefulness of healthcare professionals in incorporating new tools into their work. One could also say: they learn to understand the limits of technology, what it can and cannot do, and they adopt new behaviors, including a new sense of when their assistance might be needed. They develop a sensitivity for how care is best delivered, both with and in spite of new technology. This interaction between the nurse, the patient, and the telecare gives rise to a type of care with new practices and norms.

What has, however, not yet been addressed in her work is that the decision-making process also involves a mutual influence process. These writings focus on how nurses take action based on their understanding and intuition regarding the technology. In my cases, I demonstrate how the technology also optimizes based on feedback from humans: adjustments are made continuously. As a result, it becomes truly difficult to determine who contributes what in the decision-making process. As has been noted by Savolainen and Ruckenstein (2024), both humans and machines continuously self-correct and modify their actions in response to each other's signals and stimuli, and hence it seems they are working as one.

## Methodology and ethics

This paper is based on two overlapping research projects. The first was a preliminary investigation into the digitization of healthcare in the Netherlands, Estonia, Israel, and the UK. This research involved literature reviews conducted by myself and an assistant, as well as fieldwork that I carried out. This research took place from 2021 to 2022 and transitioned into an ERC Starting Grant for the project HEALTH-AI, which will continue until 2028 and conducts ethnographic research in six countries. The focus of this project is on the collaboration between doctors and algorithms (both AI and simpler, more protocol-driven algorithms). In this paper, I utilize the ethnographic data that I collected in two hospitals in the Netherlands, and two in Estonia. To conduct anthropological fieldwork in medical settings can be challenging as it involves sensitive, private knowledge of both patients and caretakers, which is why it is relatively uncommon. In this case, however, the focus of this research was not on the patients, but solely on their professional caretakers. Nonetheless, caution was necessary, and it was crucial to consider ethical issues. For example, what to do if I would have discovered that a decision made by a doctor in collaboration with, or based on, an algorithm, resulted in an error or harm to a patient? Due to the sensitivity of the research, I have worked with selected ethical advisors in all countries. The main methods used during the fieldwork were semi-structured in-depth interviews with medical experts, numerous informal conversations with key individuals in the public health domain, as well as participant observation in the offices, canteens, and staff rooms of medical centres where professional caretakers work. While the formal interviews provided insightful data, it was during the informal conversations that trust was established between us, as researchers, and the practitioners involved. Ethnographic data supplemented literature research, analysis of newspaper clippings, and relevant podcast recordings. Interviewees were selected using the ‘snowballing’ technique: initial participants were asked to recommend other influential individuals in the health field, leading to introductions to new interviewees through them, which facilitated access. To protect the privacy and well-being of my research participants, I anonymized their names and omitted important details such as the hospital where they work. Almost all interviews were recorded on audio; if an interviewee was uncomfortable with this, researchers in our team took hand-written notes during the interview and provided additional observations and memories into audio recordings immediately after. All data were stored in coded and anonymized formats on secure laptops and analyzed using the qualitative software program Atlas.ti.

## Findings

### Case study 1: algorithms as digital colleagues in the Netherlands

The dermatologist (47 years old, male) turned his office chair around to show me exactly how it worked, the algorithm that could diagnose skin cancer based on photos of moles. We were sitting next to each other, both on office chairs and in front of the computer in his office, in a Dutch hospital. The majority of the screen displayed a photo that was unrecognizable to me as a layperson, but to him, an expert, it was a clear example of a non-dangerous mole. “And that is confirmed by the algorithm, as you can see from the low risk percentage displayed here,” he pointed out. Indeed, on the right side of the screen, in small numbers, there was a figure with a zero before the decimal point. "From what percentage does it mean there is a high risk of skin cancer, so when do you know it is better to remove the mole surgically or start another treatment?" I asked the dermatologist. My question stemmed from the information I had received about this algorithm from the software engineers who designed it. They praised the algorithm for its accurate predictions, which were endorsed by several doctors who extensively used this AI: it was rare that the algorithm did not recognize a case of skin cancer. Additionally, one of the advantages of this product was its clear guidance to the doctor: the app used round numbers and colors—just like the AI used by the cardiologist mentioned earlier in this article, where red signaled an alarm, and green meant the doctor did not need to worry about the patient in question. I had therefore expected the dermatologist to give me a number, or even a color, but instead, he gave me a negative reply: “No, that’s not how it works in practice. The computer gives me an alarm signal if the number is extremely high, and that is when my work truly begins, as I investigate whether that alarming signal is valid or if the system has simply gone haywire.” Two points demand a brief explanation here. First, the word 'investigate' suggests that this clinician approaches the situation rationally and mathematically to determine whether an intervention is necessary for this patient. However, this idea overlooks the intuition and an inexplicable sense of ‘knowing’ that underlies much of medical knowledge and that is also acknowledged by this particular clinician and his colleague-informants in my places of fieldwork—I offer examples below. Second, the words ‘has gone haywire’ in his last sentence alluded to a complaint I had heard frequently about this algorithm. Because while it was rare for the AI to miss recognizing skin cancer in a mole, pilot studies indicated that it often mistakenly sounded false alarms: these so-called ‘false positives’ were unnecessary stressors for the patient and frustrating for the clinicians. Importantly, the dermatologists I encountered in the field were familiar with these varying results from pilot studies and seemed to have developed a strategy to deal with them. They did not blindly follow the AI's advice but instead combined the intelligence provided by the AI with their gut feeling, often described as the “gut feeling” or intuition that experienced doctors develop through years of practice. My findings in the field align with what has been written by other scholars on this type of AI designed to identify cancer in moles. Research by Tschandl et al. (2020) found that particularly experienced doctors often go against the AI's recommendations because they trust their gut feeling as much, if not more, than the risk calculations provided by the AI. From my interviews, I concluded that this did not mean they did not take the AI’s advice seriously, let alone that there was distrust in the algorithm. On the contrary, they found it to work extremely well most of the time but also considered their own expertise equally important. They seemed to ‘sense’ when an advice should not be blindly followed, double-checking the outcome in their own words. None of them were able to explain to me when they knew they should not follow a result from the algorithm. “You just know,” said a female doctor who had graduated from medical training a little over 3 years and now worked in a Dutch hospital, and another, a middle-aged clinician who works in a hospital in Israel, stated, “it’s not even a decision; it’s more that sometimes you realize you are not satisfied with what the AI says, while other times you assume the result is correct without any doubts.”

The dermatologist I was sitting with in the office in the Netherlands did not explain his approach in words, but rather through body language: when he showed me a result where the number on the screen was high, the colors alarmingly red, he narrowed his eyes, shook his head, and zoomed in on the photo to examine it more closely. “There’s something off here,” he said, “I want to see that patient in person before taking any action without further consideration.” He did not know exactly why, but he knew it had to be that way. In these examples, the 'mechanical sixth sense' described by Pols in her study of nurses collaborating with telecare is clearly evident. It is also apparent that the clinicians in my research do not simply adhere to rigid protocols or rely solely on the outcomes produced by their nonhuman colleague (the AI). Instead, they engage in a process of 'collaborative tinkering' with the AI: for each patient, this human–nonhuman decision-making hybrid adjusts its approach to what the caregivers believe to be the best course of care. Quality care varies from patient to patient and moment to moment. However, my analysis goes a step further than Pols’ narratives about the actions of nurses after the introduction of new technology. The examples discussed in this paper—both about the nurse, and the cardiologist—underscore that decisions are not merely made by either the physician or the machine. Rather, it is a collaboration with AI in which physicians develop the skill to discern when to follow AI advice blindly and when to rely on their intuition, honed through human experience. This collaboration is reciprocal; it is a dynamic, ongoing, and fluid process. The clinicians in the presented case study learn from each interaction with AI. For instance, after calling in a patient they suspect may be at risk—based on AI outcomes and their own intuition and expertise—if they later discover that the patient is perfectly healthy, their trust in AI may diminish, and their intuition could be affected as well. Conversely, their feedback into the AI system will contribute to its optimization for future use. This means that, in a sense similar to the physician, the AI adjusts progressively, and decisions are made by what can only be described as a hybrid, decision-making unit. In this unit, agency and intelligence are distributed, and information is constantly exchanged between human and machine, a dynamic that only ends when a saturated result is reached. This unit, and the interaction between the actors, was not only evident from interviews but also from the observed movements and behaviors of professional caretakers towards the algorithms they work with. Physicians never spoke about the algorithm as if it were a soulless thing but as an actor with agency, and often even as a valued colleague. “This is my thinking buddy,” said the Dutch female dermatologist about the algorithm, and the cardiologist referred to the AI he worked with as “an indispensable sparring partner.” Physicians often leaned towards the computer screen to read something or spoke to it aloud: “well, I don’t believe you,” they would say if they suspected the algorithm was giving a false alarm, or “oh, that does not look good indeed,” if the algorithm managed to draw their attention to a high-risk case. If I had heard those words without seeing the image in front of me, I would have thought the physicians were talking to human colleagues. Whereas the physician’s intuition was previously grounded in knowledge acquired from books and mentors, combined with experience gained from daily work, it appears that a third factor has now been incorporated into their decision-making: data provided by an algorithm. In this interactive collaborative decision-making process, the physician and the machine evolve together as a unit that functions increasingly effectively (Savolainen and Ruckenstein 2024).

I will provide a second case example from the field because it shows a very different, important side of how collaboration between human and machine can look like in the daily work of professional caretakers—a side that is concerning as it raises new ethical questions that have not yet been answered, while reality is catching up with us.

### Case study 2: flawless algorithms in Estonia

Several weeks, I conducted fieldwork in Estonia, to interview physicians and medical researchers specializing in rare population diseases. In different labs, AI was used to better understand the factors leading to these rare diseases; my findings concern one of those labs, which I keep anonymous to protect the privacy of my informants. In this lab, a particular AI system searched for trends and patterns in blood samples: for example, individuals with a certain ethnic background appeared to be more vulnerable to disease X, and those with certain high levels in their blood were more likely to develop condition Y. The AI worked so quickly and seemingly effectively, with such big data sets, that no human could have ever replicated it. It would even be fair to say that it was doing a newly invented job, a job that only existed since this particular AI had been built. Since the invention of this algorithm, more rare diseases have been detected in Estonia than before, according to the physicians in the department. As did the clinicians I met in Dutch and Israeli hospitals, they too spoke with affection and pride about the algorithm with which they worked. However, in this particular country case example, I found little evidence of a balanced interaction between the physician and the algorithm. All interviewees showed to be aware of the importance of having humans in the loop in any Ai system, and indeed, they would emphasize their role as that of the human overseer on the floor, saying things like “the AI should never work autonomously, it is here to support our work”, and “this AI is like an extension of our own brains”. Hence, formally, in this case, too, there was human oversight; a human in the loop system in which a human monitors, and evaluates, the Ai. However, in practice, it seemed to me that the AI was doing the work and the human caretakers were following it patterns.

All the physicians I met there indicated that they trusted every result generated by the AI. Without exception, my interviewees would describe it as completely trustworthy: trained on large amounts of data, having been in use for several years and never caught making a mistake. However, none of them understood how the algorithm was built, on how much data it was trained exactly, what data it specifically used to discover patterns in its data, or how it arrived at its predictions. Admittedly, this would be an unfair expectation of physicians who are trained in medical expertise, after all, not programming. But it did mean that the physicians were unable to utilize, or develop, a mechanical sixth sense. Whatever the Ai did could not be checked by the employees in these medical institutions, first because the AI was taking up a job that human doctors never had done, and never would be able to do. Second, AI was not effectively oversighted by humans because clinicians seemed no longer seemed aware of the fact that, of course, there is no such thing as ‘neutral’ data to begin with. As is common knowledge in social science and data studies, technology, including algorithms, is constructed from political choices (Pfaffenberger 1992). The programmer chose to exclude certain factors from the design, not to weigh certain data, or to include it explicitly. Nevertheless, the human clinicians working with the machine described it as flawless, or in the exact words of the department head when I asked whether the AI ever made a mistake: “What do you mean? This algorithm makes no mistakes! [laughs]That is impossible. Only us, [informant points index finger at their own chest] humans make mistakes. Never trust us, always trust the machine. [laughs]”.

Anthropologists conduct research based on the belief that people do not always express in formal interviews what they actually believe or do; this is one of the main reasons why observations and informal conversations are an important part of our methodology. However, in this case, I was unable to discern any difference between the trust narrative and the actions of my respondents. I was only allowed to attend one, brief meeting where the outcomes of AI were discussed by clinicians and PhD researchers, but what I observed there was affirmed by what I also witnessed during informal meetings among staff members (such as coffee breaks and birthday parties in the canteen), where results and other work-related matters were discussed. In these meetings, the outcome of an AI-generated calculation was typically taken as a fact and presented as a starting point for applying for a new research grant, initiating new research, or writing an article. No one in the room questioned whether the calculation was accurate, whether a check had been conducted, or what data or initial assumptions the calculation was based on. One day, waiting for an interview with one of the senior medics, I wondered around in the offices where staff members were working with the AI. It was my impression that the atmosphere in the offices was one of hard work, but also enjoyable: when someone had a birthday, it was standard to celebrate with cake and coffee in the staff kitchen. The staff appeared mostly young and had an international background—many doctors came to work in Estonia while simultaneously conducting PhD research, the head of the department proudly told me. They were drawn to the country and this institution because of its reputation for being at the forefront of AI in healthcare. When I asked these young researchers how they specifically worked with AI, they consistently pointed to a computer screen that grouped numbers: “Look, you can clearly see a pattern here”, they would say, or “see, the AI shows that this disease seems to be more prevalent amongst this part of the population.” They told me that their task was to propose new research or write a new article based on these findings. No one ever mentioned evaluating or critically assessing the outcomes—these were seen as the starting point, not as a part of their work that needed scrutiny. When I asked one informant if someone should serve as the ‘human overseer,’ she exclaimed in surprise, “But how could that be? I have no idea what this AI exactly does! But it's a really good system, I assure you, it never makes mistakes.”

I do not claim that this particular algorithm made errors (nor do I know sufficiently about the workings of AI to even try and guestimate its effectiveness), but I do propose that nevertheless, there is reason for human collaborators of the AI to critically evaluate certain outcomes. While the algorithms themselves may not make “mistakes” in the traditional sense of the word, many scholars have shown that they certainly yield distorted or biased results due to the way they are designed, trained, or deployed (Carboni et al. 2024; Osoba et al. 2017; Buolamwini 2023; Noble 2018; Saxena 2023; Katz 2020), or wrote about related ethical issues (Coeckelbergh 2020; Dubber 2020; Govia 2020; Liao 2020; Zuboff 2019; O’Neil 2016).

In this particular case in one lab in Estonia, it turned out that the programmer who built the algorithm as part of his PhD research, was funded by a pharmaceutical company. He obtained his grade several years ago in the institute. The company funding him had asked for an algorithm that could detect certain diseases—not unsurprisingly, these were diseases that they either had, or were developing, medicine and treatments for. Hence, it was not (just) doctors, but a pharmaceutical company deciding that certain diseases would be detected by the algorithm, while others would not. Yet, in the daily practice of work, the clinicians nowadays utilizing the AI seemed to have forgotten, or were seemingly oblivious about its political history. Transparency and ethical scrutiny of the use of AI in the healthcare sector are essential to safeguard the integrity and reliability of healthcare. The discussion, below, elaborates on this point.

## Discussion

My analysis critiques “the emphasis on the individual—whether human or machinic—as the primary source of intelligence the central unit of ethical deliberation” (Rella and Lapaolo forthcoming: LL17-18). The findings presented in this paper raise important implications for our understanding of ethical AI. Hayles cautions against the lack of ethical frameworks suited for cognitive assemblages (2022: 1195). She identifies several ethical concerns, including accountability when algorithms are involved, the specific harms associated with cognitive assemblages, methods for mitigation, the potential for programming algorithms to adopt ethical norms and behaviors, and whether computational agents themselves deserve ethical consideration. Hayles emphasizes the urgent need for developing ethical frameworks for cognitive assemblages. While establishing such frameworks goes beyond the scope of this paper, I will highlight specific ethical issues and questions that emerged during my anthropological fieldwork in hospitals in the following discussion. The introduction of this paper already posited that the current approach to contemplating, and particularly formulating rules for, so-called ethical AI is problematic. In this section, I aim to further explore this assertion, with a particular focus on the concept of Human in the Loop (HITL) and, even more precise, the human clinician who formally functions as human overseer of the AI—the person who is supposed to check AI outcomes, and resist when necessary. As noted, AI regulations currently maintain that humans must be actively engaged in the decision-making process of these systems, in order to ensure a non-biased, non-flawed, ethical outcome. The idea of a human overseer conjures the image of two actors, working detached from each other: first the computer, and then, for validation, the human. However, my own ethnographic findings, as well as work of other scholars discussed, demonstrate that this line of thinking is not backed by the daily practice of the work of professional caretakers collaborating with AI. In all the examples I provided from my fieldwork in hospitals, there certainly was an appointed human overseer present whose role was to interpret the data and outcomes and, if necessary, identify or resist errors. This is also the case for nurses with whom Jeannette Pols conducted fieldwork, whose research centered on nurses in the context of new technology, who have the responsibility to provide ‘good care’ on the basis of data. Yet, the human caretakers we encountered in our fields did not emerge as actors who made decisions separately from the technology they utilized. In contrast, human actors interact in a constant cognitive assemblage (Hayles 2022): a dynamic with algorithms, data, ideas, thoughts, reflections, and intuition are ongoingly exchanged, created and amended, together. Based on this ‘tinkering’ dynamic, a decision emerges, but it is very difficult to discern exactly who made it and why. My best answer to that question would be: by the hybrid decision-making unit of human and machine. Would the human and the AI have worked completely separately from each other, they would likely have come up with different decisions than in this hybrid partnership. This paper does *not* contradict the notion that both humans and machines possess different capacities which we could label intelligence, but rather emphasizes the intricate interplay between them. They engage in what I term ‘collaborative tinkering,' where their decisions emerge from a dynamic and continuous interaction, with each influencing the other through feedback on the outcomes they produce. This collaborative process involves both rational knowledge and intuitive insight, revealing the multifaceted nature of decision-making in medical settings. This suggests that the notion of a human making decisions in isolation from the algorithm with which they collaborate is simplistic, if not unrealistic. In the realm of healthcare, professional caregivers form hybrids with their ‘digital colleagues’, and decisions are made by this hybrid unit through ongoing interactions in what Hayes might call cognitive assemblages (2022: 1195): between humans, machines, and the environment in which they operate. Hence, professional caretakers and algorithms interact as hybrid decision-makers, and the concept “tinkering” applies to their unison, as it is in their collaboration that human and algorithm experiment and adjust to develop effective care practices. This argument, which highlights the complex and intertwined nature of decision-making between humans and machines, has implications for the prevailing emphasis on the human-in-the-loop in regulations and frameworks around ethical AI. The first case presented in this paper shows that common terms such as ‘human in the loop’, or ‘human overseers’, risk to provide a false picture of how decision-making works in the daily practice of human–nonhuman collaboration. The second case presented in this paper sketched a more concerning picture: it made clear that, even if the clinicians are formally considered the human overseer of Ai and recognize this role as important, in practice, they appeared unable to check or correct for potential errors of the AI: its working were too opaque. To some extent, here too, human and nonhumans were considered a hybrid, decision-making unit (hence the remarks by clinicians about AI being an ‘extension’ of their own brain). However, in this case, there seemed to be little evidence of tinkering or a mechanical sixth sense. This is most likely related to the fact that these clinicians rarely saw individual patients; they were observing numbers on their screens and, as a result, were unable to develop their intuition and experiential expertise. In this sense, it is not a fair comparison of cases; nonetheless, I consider it important to discuss this case because it illustrates so painfully how the practice of working with AI often diverges from the narrative surrounding it, as well as the regulations concerning ethical AI. Hence, an additional problem for our understanding what ‘ethical AI’ means, is that the emphasis on the human overseer creates a false sense of security in the context of healthcare, especially considering that clinicians or nurses may not have a specialized background in software programming or a deep understanding of AI. This places a significant ethical burden on individual clinicians, as not all of them may have the ability to question AI outcomes or fully comprehend the complexities of AI algorithms. As the ethnographic cases showed, often, doctors or nurses may not exactly rationally know why they do or do not fully trust certain outcomes, but ultimately make a decision that is partly based on algorithmic results and partly on their own experience, knowledge, or intuition (case 1, in the Netherlands) or on their trust in AI (case 2, in Estonia). This highlights the complex interplay between human judgment and AI-driven outputs in the decision-making process, and underscores the urgent need for re-evaluating AI ethics.

Clearly, it is crucial that healthcare professionals remain aware of the limitations and any potential biases in the algorithms they use, and that they continue to critically reflect on the interpretation and application of the results. To put that differently: the agency and intelligence between humans and machines must remain somewhat distributed and balanced in collaborative tinkering. This was the case in my first case in the Netherlands, presented in this paper. It was also the case in the work of Jeannette Pols, where nurses were developing a ‘mechanical sixth sense’ to sense, intuitively, whether technology was providing outcomes that were correct, or off. A similar critical or adaptive type of collaboration was described recently in a paper by Carboni et al. (2024). They studied a trial of an algorithm predicting inpatient violence in two Dutch psychiatric clinics, and found that while nurses approached violence assessment with caution, striving to avoid unnecessary punishment of patients, the algorithm promoted a preemptive and potentially punitive stance. This contrast raises serious concerns about the ethical implications of incorporating such algorithmic outputs into decision-making in sensitive situations. However, the paper also left me somewhat hopeful, as it became clear from the research that the nurses did not simply accept the outcomes produced by the AI. Instead, they interacted with the algorithmic scores by trying to bring some doubt back into them. Whether they succeed or not, the article does not elaborate on, but their resistance, to me, is an example of how professional caretakers continually tinker; how they improvise, intuitively sense what is going on with a patient, and how they feed that embodied expertise back into the AI.

These examples indicate that it is important that clinicians continue to provide feedback into AI systems and not automatically go along with the decisions presented by the algorithm (as happened in the lab). Developing a mechanical sixth sense can assist with this, but to do so, it is necessary to first cultivate one's own intuition based on experience and practice. This is not feasible in a lab as discussed in the second case, where young doctors have never had to perform a task independently before immediately starting to work with AI. This also illustrates that, in the context of AI that operates based on big data (as was the case in the place where I conducted fieldwork), it is a naive notion to believe that doctors will assume a ‘human overseer’ role, especially since humans are significantly less adept at recognizing patterns within large datasets. Nonetheless, this is currently reflected in the legislation and echoed within healthcare institutions. If, as seems the case with this particular case in Estonia, it is impossible for clinicians to understand the working of AI due to its complexity, it should be more clear in regulations and expectations that clinicians should not serve as human overseer, and we need to think about who, then, should take on this job.

## Conclusion

Insights into the hybrid nature of human–computer collaboration are essential for understanding the computational practices of our time, as they challenge and disrupt preconceived notions about both our technologies and ourselves. Instead of solely focusing on human agency within human–machine systems and viewing intelligence as confined to individuals, whether human or machine, this paper adopted the perspective that agency and intelligence are distributed across various entities, including social and computational realms. Viewing humans and machines as a single hybrid decision-maker encourages us to see distributed agency as a foundational concept for reimagining AI ethics. As the editors of the Special Issue highlight, “as human and machine decisions become increasingly intertwined, it is crucial to envision new ethical and political responses that move beyond traditional individualistic viewpoints.” This paper aligns with, and builds upon that view, in exploring questions around intelligence in situations where humans and AI collaboratively make decisions, in the realm of healthcare. Proposing that the decision-making occurs not from ‘ethical AI’, with an autonomous human in the loop, but instead from a human–nonhuman, decision-making hybrid, the paper challenges the emphasis on the individual—be it human or machinic—as the primary locus of intelligence and the fundamental unit of ethico-political concern. Instead, it embraces the provocations that intelligence is always “distributed across human and technical agencies” (Amoore 2020, p. 4), and that agency is distributed over the material and the nonmaterial (Callon and Law 1995). In the context of healthcare, the concept of physicians as the 'human in the loop' mandated by legislative requirements for 'ethical AI' is shown to be somewhat misleading. This highlights the need for a deeper understanding of the dynamics between humans and AI, as well as the recognition of the limitations and challenges that come with such partnerships in healthcare and beyond. Therefore, this paper calls for more research that studies the daily practices of people and machines, from an ethnographic perspective, rather than the regulations around AI, or the design of AI. Clearly, the interaction between humans and machines in decision-making processes is far more nuanced and complex than simply inserting a human in the loop for ethical considerations—the ethnographic examples from my fieldwork showed that human clinicians, in the interaction with the AI, are constantly involved in a tinkering dynamic of an exchange of knowledge and experience, which leads to intuitions and decisions. This fusion, or the decision-making hybrid as it is called in this article, illustrates the intricate and nuanced nature of the human–AI partnership in healthcare, where both elements play crucial roles in ensuring the best possible outcomes for patients. However, it was also shown that such collaborative tinkering can only arise in situations where humans (somewhat) understand the AI system they are working with, and perhaps even more importantly, get to see patients rather than their data on a screen. Only in these human interactions, intuition and bodily experience can develop; the same goes for a mechanical sixth sense. At least two pathways for future research can be suggested. One is to engage empirically with the theme of human–nonhuman collaboration, either in healthcare or other fields, to see how regulations around ethical AI, or expectations about how people work with technology, unfold in daily work. Another is to further look into the observed difference between clinicians working with AI in the context of encounters with patients, versus clinicians who work merely with big data and thus have to lean on AI calculations that are hard for them to understand and check.

## Data Availability

The raw and processed data are protected and are not available due to data privacy laws. This is common practice in ethnographic research, as to protect informants. Oral informed consent was obtained from all participants, and ethical approval for the research was provided by the Amsterdam Institute for Social Science Research (AISSR) and University of Amsterdam’s Ethical board.
